# Paraoxonase 1 Suppresses Hepatocellular Carcinoma Progression by Modulating the NOD-like Receptor Signaling Pathway

**DOI:** 10.3390/biom16060774

**Published:** 2026-05-25

**Authors:** Hao-Lin Wei, Zhi-Ce Tan, Chu-Heng Gou, Ling Wang, Ze-Kun Liu, Hui-Jie Bian

**Affiliations:** 1 Department of Cell Biology, National Translational Science Center for Molecular Medicine, Fourth Military Medical University, Xi’an 710032, China; 2 State Key Laboratory of New Targets Discovery and Drug Development for Major Diseases, Xi’an 710032, China; 3 Department of Hepatobiliary Surgery, Xijing Hospital, Fourth Military Medical University, Xi’an 710032, China; 4 Department of Health Statistics, School of Preventive Medicine, Fourth Military Medical University, Xi’an 710032, China

**Keywords:** paraoxonase 1, hepatocellular carcinoma, prognostic biomarker, tumor suppressor factor

## Abstract

Hepatocellular carcinoma (HCC) represents one of the most lethal malignancies worldwide and is characterized by profound metabolic reprogramming during its development. Paraoxonase 1 (PON1), a liver-synthesized secretory protein involved in lipid metabolism, has an incompletely defined role in cancer biology. This study aimed to systematically investigate the expression pattern, clinical features, and biological function of *PON1* in HCC through an integrated approach combining data mining, RNA-seq and experimental verification. Our results demonstrated that *PON1* expression is significantly downregulated in HCC tissues compared with adjacent tissues. Clinically, significant disparities were observed in gender (χ^2^ = 19.305, *p* < 0.0001), tumor stage (χ^2^ = 18.030, *p* = 0.0004), and tumor grade (χ^2^ = 13.391, *p* = 0.0039) between patients with high and low *PON1* expression in HCC. Low *PON1* expression was associated with poor prognosis (TCGA_LIHC, log-rank: χ^2^ = 9.290, *p* = 0.0023; ICGC_LIRI, log-rank: χ^2^ = 8.469, *p* = 0.0036; GSE14520, log-rank: χ^2^ = 9.746, *p* = 0.0018). Univariate and multivariate Cox regression analyses revealed *PON1* as an independent prognostic biomarker. Pathway analysis showed that *PON1*-positively correlated genes enriched in pathways such as peroxisome and fatty acid degradation, whereas *PON1*-negatively correlated genes mainly in the cell cycle pathway. Functional experiments confirmed that knockdown of *PON1* promoted HCC cell proliferation, migration, invasion and inhibited apoptosis, whereas overexpression of *PON1* reversed these malignant phenotypes. Mechanistically, we uncovered that PON1 exerts its tumor-suppressive effects by negatively regulating TANK and CXCL3, key molecules of the NOD-like receptor signaling pathway. In summary, our findings identify PON1 as an independent prognostic biomarker in HCC and demonstrate the tumor-suppressive role of PON1, indicating its potential as a therapeutic target for HCC.

## 1. Introduction

Hepatocellular carcinoma (HCC) remains a major global health challenge, characterized by both high incidence and high mortality rates [1]. Although treatment options such as surgical resection, liver transplantation, and local ablation are available for early-stage disease, and the majority of patients are diagnosed at advanced stages. Furthermore, responses to systemic therapies, such as multi-kinase inhibitors and immune checkpoint inhibitors, are markedly difference, and acquired resistance poses a persistent clinical challenge [2,3]. Consequently, a deeper dissection of the molecular drivers of HCC progression, coupled with the identification of novel prognostic biomarkers and therapeutic targets, remains a critical unmet need in both clinical and translational research.

Paraoxonase 1 (PON1) is a calcium-dependent esterase/lactonase primarily synthesized in the liver and secreted into the circulation, where it associates with high-density lipoprotein (HDL) and hydrolyzes oxidized phospholipids and aromatic esters, thereby exerting antioxidant, anti-inflammatory, and cardioprotective effects [4,5]. The functions of PON1 vary across different tumors, and its role is highly dependent on specific cell and tissue types. For instance, it may act as an oncogene by promoting cell proliferation and metastasis in colorectal and lung cancers [6,7], yet exhibit tumor-suppressive activity in breast cancer [8]. In addition, elevated PON1 expression conferred protection against nonalcoholic fatty liver disease by reducing oxidative stress [9].

PON1 has been implicated in HCC as a prognostic biomarker and predictor of microvascular invasion [10,11]. Bioinformatics analyses also linked low *PON1* expression to poor prognosis [12]. Moreover, Lu et al. revealed that *PON1* suppressed HCC via VHL-mediated HIF-1α degradation, reducing lactate and Treg infiltration [13]. However, the clinical significance of *PON1* has not been systematically analyzed, and its biological functions in HCC cells as well as the underlying molecular mechanism of its tumor-suppressive function have not been fully elucidated.

In this study, we aimed to systematically elucidate the expression pattern and clinical significance of *PON1* in HCC using the multi-omics database. Moreover, we performed the loss-of-function and gain-of-function assays to explore the potential roles of *PON1* in HCC. Finally, we explored the potential tumor-suppressing mechanism of *PON1* in HCC using RNA-seq. Our findings highlight the potential of targeting PON1 as a therapeutic strategy in HCC.

## 2. Materials and Methods

### 2.1. Public Data Analysis

Pan-cancer and HCC transcriptome data (quantified as log_2_(TPM + 1)), clinicopathological information, and survival data were obtained from The Cancer Genome Atlas (TCGA) database [14,15]. Independent validation datasets were sourced from the International Cancer Genome Consortium (ICGC) LIRI-JP cohort and the Gene Expression Omnibus (GEO) dataset GSE14520. Proteomics data (quantified as Z-Value) were retrieved from the Clinical Proteomic Tumor Analysis Consortium (CPTAC) [16]. Cell line expression data were referenced from the Human Protein Atlas (HPA) cell line [17] and the Cancer Cell Line Encyclopedia (CCLE) [18].

### 2.2. Tumor Immune Infiltration

Immune cell infiltration scores within the tumor microenvironment were calculated using CIBERSORT (https://cibersortx.stanford.edu/) [19]. Correlation analyses between immune cell abundance and gene expression or copy number variation status were performed using TIMER 2.0 (http://timer.cistrome.org/) [20].

### 2.3. Protein–Protein Interaction and Correlation Analysis

Protein–protein interaction networks were predicted using GeneMANIA (http://www.genemania.org) [21]. Analysis of genes correlated with *PON1* and functional enrichment analyses were carried out using LinkedOmics (http://www.linkedomics.org) [22]. KEGG and GO enrichment analyses were conducted using the clusterProfiler (v4.18.3) in R [23].

### 2.4. Cell Culture and Transfection

Human HCC cell lines (MHCC97H, Huh-7, Hep3B, HepG2, HCCLM3, Sun387, and Sun182) were purchased from ATCC (Manassas, VA, USA) or the Chinese Academy of Sciences Cell Bank (Shanghai, China) and cultured in RPMI-1640 medium supplemented with 10% fetal bovine serum at 37 °C under 5% CO_2_. For loss-of-function studies, *PON1*-specific siRNAs (si-319, si-471, and si-775) were purchased from GenePharma (Suzhou, China) and transfected into high-expressing Huh-7 cells using Lipofectamine 2000 (Mei5 Biotechnology Co., Ltd, Beijing, China). For gain-of-function studies, the full-length human *PON1* coding sequence was cloned into a lentiviral overexpression vector and used to infect low-expressing HCCLM3 cells. HCC cells with stable *PON1* overexpression (*PON1*-OE) and negative control (NC) were screened using puromycin.

### 2.5. Cell Functional Assays

Cell proliferation was assessed using the CCK-8 assay. Cell migration and invasion capacities were evaluated using Transwell chambers; for invasion assays, chambers were pre-coated with Matrigel. Cells that migrated or invaded to the lower chamber were counted under a Leica inverted microscope (Leica Microsystems, Wetzlar, Germany) from five randomly selected fields. Apoptosis was detected using Annexin V-FITC/PI or Annexin V-APC/7-AAD kits (KeyGEN BioTECH, Nanjing, China) via a BD FACSCalibur flow cytometry (BD Biosciences, San Jose, CA, USA) and analyzed using FlowJo (v10) software. Cell cycle distribution was analyzed by PI/RNase A staining, and the proportion of cells in each phase was determined using ModFit LT (v5.0) software.

### 2.6. Quantitative Real-Time PCR (qRT–PCR)

The protocol used for qRT–PCR was described in our previous publication [24]. The primer sequences were as follows: *PON1*: forward primer 5′-CTGATTGCGCTCACCCTCTT-3′, reverse primer 5′-CGGAGAGCATTAAGTCGTGTTTG-3′; *ACTB*: forward primer 5′-AGCGAGCATCCCCCAAAGTT-3′, reverse primer 5′-GGGCACGAAGGCTCATCATT-3′.

### 2.7. Western Blot

The protocol used for Western blot was described in our previous publication [24]. The PON1 (18155-1-AP), TANK (27065-1-AP), CXCL3 (11221-1-AP) and α-tubulin (11224-1-AP) were purchased from Proteintech (Wuhan, China).

### 2.8. Transcriptome Sequencing

Transcriptome library construction and sequencing were performed by GENE DENOVO company (Guangzhou, China). Differential expression analysis was performed using the DESeq2 software package (v1.51.6) [25], with thresholds set at |log_2_ fold change (log_2_FC) | > 0.585 and adjusted *p* < 0.05.

### 2.9. Statistical Analysis

Student’s *t*-test was used for comparisons between two groups, and one-way analysis of variance (ANOVA) followed by Tukey’s post hoc test was used for multiple comparisons among three or more groups. Survival differences were analyzed by the log-rank test. Spearman analysis was used to assess correlations between *PON1* expression and immune cell infiltration, and Pearson analysis was performed to evaluate the associations between *PON1* expression and other gene expression levels in TCGA_LIHC tumor samples. The association between the expression of *PON1* and clinical features was computed using the chi (χ^2^)-square test (abbreviated as χ^2^). After skewness in the immune infiltration abundances was corrected and variance inflation factor (VIF) tests were performed for all variables, those with VIF > 5 were removed to avoid multicollinearity [26]. Subsequently, univariate and multivariate Cox regression analyses were used to assess the association of *PON1* mRNA expression with other factors, including age, gender, grade, stage, and the relative infiltrative abundances of key immune cells (B cell, CD4^+^ T cell, CD8^+^ T cell, macrophage and neutrophil). All charts and graphs were generated using GraphPad Prism (v9.0) [27] and the ggplot2 R package (v4.0.1) [28,29]. *p* < 0.05 was considered statistically significant.

## 3. Results

### 3.1. Expression Patterns and Immune Analysis of PON1 in Pan-Cancer

**Figure 1:**
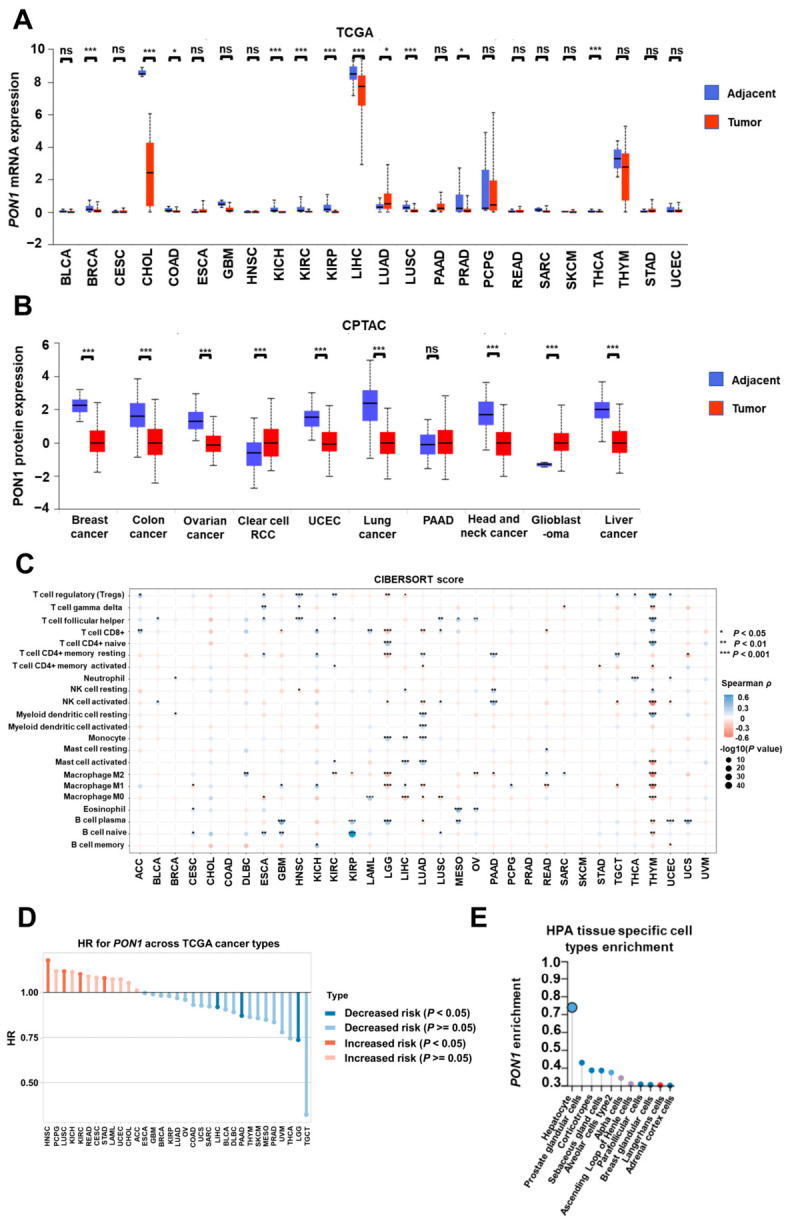
Pan-cancer analysis of *PON1* expression and immune analysis. (**A**) *PON1* mRNA expression in pan-cancer based on TCGA. (**B**) PON1 protein expression in pan-cancer based on CPTAC. (**C**) Correlation between *PON1* mRNA expression and immune cell infiltration analyzed via CIBERSORT. (**D**) Forest plot of hazard ratios (HRs) for *PON1* in pan-cancer. (**E**) Tissue-specific enrichment scores for *PON1* based on the HPA database. For (**A**,**B**), Student’s *t*-test. For (**C**), the Spearman method. ns, not significant; * *p* < 0.05; ** *p* < 0.01; *** *p* < 0.001.

Our analysis of *PON1* mRNA expression levels in pan-cancer tissues showed that *PON1* expression was significantly upregulated in LUAD tumor tissues relative to adjacent tissues, but significantly downregulated in tumor tissues of BRCA, CHOL, COAD, KICH, KIRC, KIRP, LIHC, LUSC, PRAD, and THCA compared with adjacent tissues (Figure 1A). Analysis of pan-cancer protein data based on the CPTAC database showed that PON1 protein was significantly overexpressed in clear cell RCC and glioblastoma tumor tissues, while its expression was significantly reduced in breast, colon, ovarian, UCEC, lung, head and neck, and liver cancer tissues relative to adjacent tissues (Figure 1B). To investigate the relationship between *PON1* expression and tumor-infiltrating immune cells, we used the CIBERSORT algorithm to evaluate the differences in immune infiltration between the *PON1* high-expression and low-expression groups. The results showed that *PON1* expression in HCC was positively correlated with activated mast cells (Spearman *ρ* = 0.205, *p* < 0.001) and negatively correlated with Tregs (Spearman *ρ* = −0.123, *p* < 0.05) (Figure 1C). Pan-cancer survival risk analysis demonstrated that high *PON1* expression was significantly associated with an increased risk of death in HNSC, LUSC, KIRC, and STAD (all HR > 1, *p* < 0.05), whereas it predicted a decreased risk in LIHC, PAAD, and LGG (all HR < 1, *p* < 0.05) (Figure 1D). Analysis of HPA data revealed that *PON1* was highly and specifically enriched in hepatocytes under physiological conditions, with enrichment scores substantially exceeding those of prostate glandular epithelial cells, corticotropes, and other cell populations (Figure 1E). Furthermore, analysis of tumor cell line expression revealed high *PON1* expression levels in prostate and liver cancer cell lines, but minimal expression in most other cancer lines, including lung, thyroid, and hematopoietic malignancies (Figure S1). Collectively, these findings indicate that *PON1* may be an important favorable prognostic factor in HCC.

### 3.2. PON1 Is Downregulated in HCC and Associated with Favorable Prognosis

**Figure 2:**
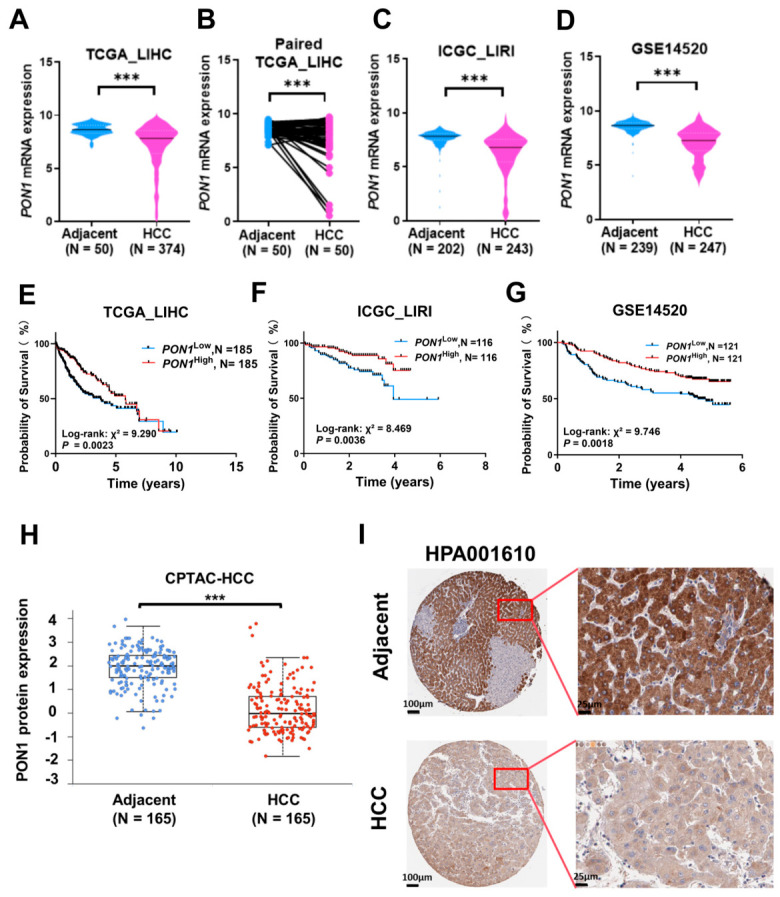
Expression and prognosis analysis of *PON1* in HCC. *PON1* mRNA expression in HCC tumor compared to adjacent tissues based on the TCGA_LIHC (**A**), TCGA_LIHC paired samples (**B**), ICGC_LIRI (**C**), and GSE14520 (**D**) datasets. Kaplan–Meier analysis of HCC patients in TCGA_LIHC (**E**), ICGC_LIRI (**F**) and GSE14520 (**G**). (**H**) PON1 protein expression in HCC and adjacent tissues from the CTPAC samples. (**I**) Representative IHC images of PON1 in HCC and adjacent tissues from the HPA database. For (**A**–**D**,**H**), Student’s *t*-test. For (**E**–**G**), log-rank test. *** *p* < 0.001.

To investigate the expression of *PON1* in HCC, we analyzed the mRNA expression of *PON1* based on TCGA_LIHC, ICGC_LIRI and the GEO dataset (GSE14520). The results consistently demonstrated that *PON1* mRNA expression was significantly downregulated in HCC tissues compared with adjacent tissues (all *p* < 0.001) (Figure 2A–D). Kaplan–Meier analysis revealed that lower expression of *PON1* was associated with shorter overall survival in three independent datasets (TCGA_LIHC, log-rank: χ^2^ = 9.290, *p* = 0.0023; ICGC_LIRI, log-rank: χ^2^ = 8.469, *p* = 0.0036; GSE14520, log-rank: χ^2^ = 9.746, *p* = 0.0018) (Figure 2E–G). Consistent with these transcriptional findings, proteomic data from CPTAC confirmed that PON1 protein expression was markedly lower in HCC tissues relative to adjacent tissues (Figure 2H). Immunohistochemical images from the HPA database corroborated this reduction in HCC tissue (Figure 2I).

### 3.3. Association Analysis of PON1 mRNA Expression with Clinical Characteristics in HCC

**Figure 3:**
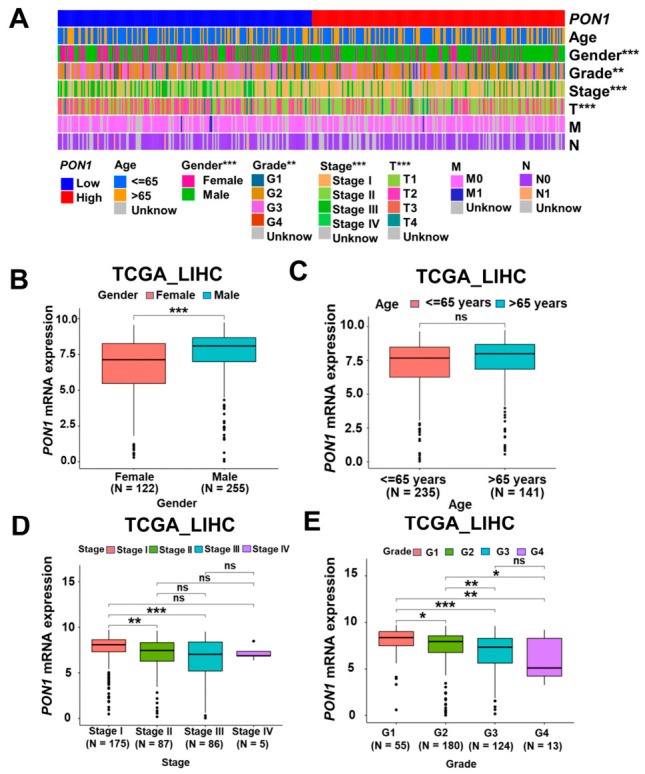
Association of *PON1* mRNA expression with clinicopathological features in HCC. (**A**) Heatmap showing the association between *PON1* mRNA expression and clinicopathological features in TCGA_LIHC. *PON1* mRNA expression in the TCGA_LIHC cohort stratified by gender (**B**), age (**C**), tumor stage (**D**), and histological grade (**E**). For (**A**), χ^2^ test. For (**B**,**C**), Student’s *t*-test. For (**D**,**E**), one-way ANOVA followed by Tukey’s post hoc test. ns, not significant; * *p* < 0.05; ** *p* < 0.01; *** *p* < 0.001.

We further analyzed the correlation between the expression of *PON1* and clinical characteristics in HCC patients. The results showed that significant disparities were observed in gender (χ^2^ = 19.305, *p* < 0.0001), tumor stage (χ^2^ = 18.030, *p* = 0.0004), and tumor grade (χ^2^ = 13.391, *p* = 0.0039) between patients with high and low *PON1* expression in HCC (Figure 3A). In addition, we analyzed the differences in *PON1* expression among groups stratified by different clinical characteristics. The results indicated that *PON1* expression was significantly higher in male patients than in females (χ^2^ = 19.305, *p* < 0.001) (Figure 3B), whereas no significant association with age (≤65 years vs. >65 years) was observed (χ^2^ = 2.600, *p* > 0.05) (Figure 3C). Notably, *PON1* mRNA expression showed a gradual decline with increasing tumor stage (Figure 3D) and tumor grade (Figure 3E).

**Figure 4:**
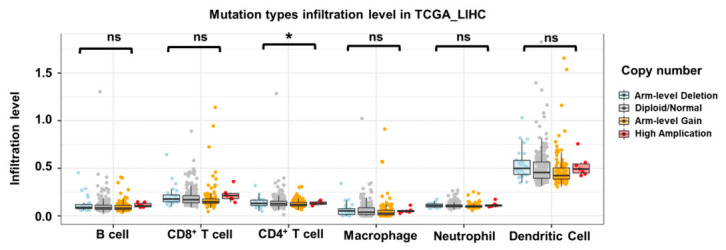
Impact of *PON1* copy number status on immune cell infiltration scores in the TCGA_LIHC dataset. One-way ANOVA was used to compare the differences between groups. ns, not significant; * *p* < 0.05.

The relationship between copy number variations (CNVs) of *PON1* and tumor immune cell infiltration was investigated. Notably, the infiltration level of CD4^+^ T cells exhibited a significant overall difference among groups stratified by *PON1* copy number status (e.g., arm-level deletion, gain, etc.) (*p* < 0.05), whereas no differences were observed for other immune cell subsets, including B cells, CD8^+^ T cells, macrophages, neutrophils, and dendritic cells (DCs) (Figure 4).

After removing DCs whose VIF values were more than 5 (Table S1), univariate and multivariate Cox regression analyses were constructed. The results showed that univariate Cox regression analysis indicated that PON1 mRNA expression (HR = 0.858, 95% CI: 0.798–0.921, *p* < 0.001) and tumor stage (HR = 1.680, 95% CI: 1.369–2.062, *p* < 0.001) were notably related to overall survival (Table 1). We further performed a multivariate Cox regression analysis and confirmed that *PON1* mRNA expression (HR = 0.846, 95% CI: 0.777–0.921, *p* < 0.001) served as an independent predictor for overall survival (Table 1). Collectively, these findings demonstrate that decreased *PON1* expression may contribute to the malignancy progress of HCC cells.

### 3.4. Protein–Protein Interaction and Correlation Genes Analysis of PON1

**Figure 5:**
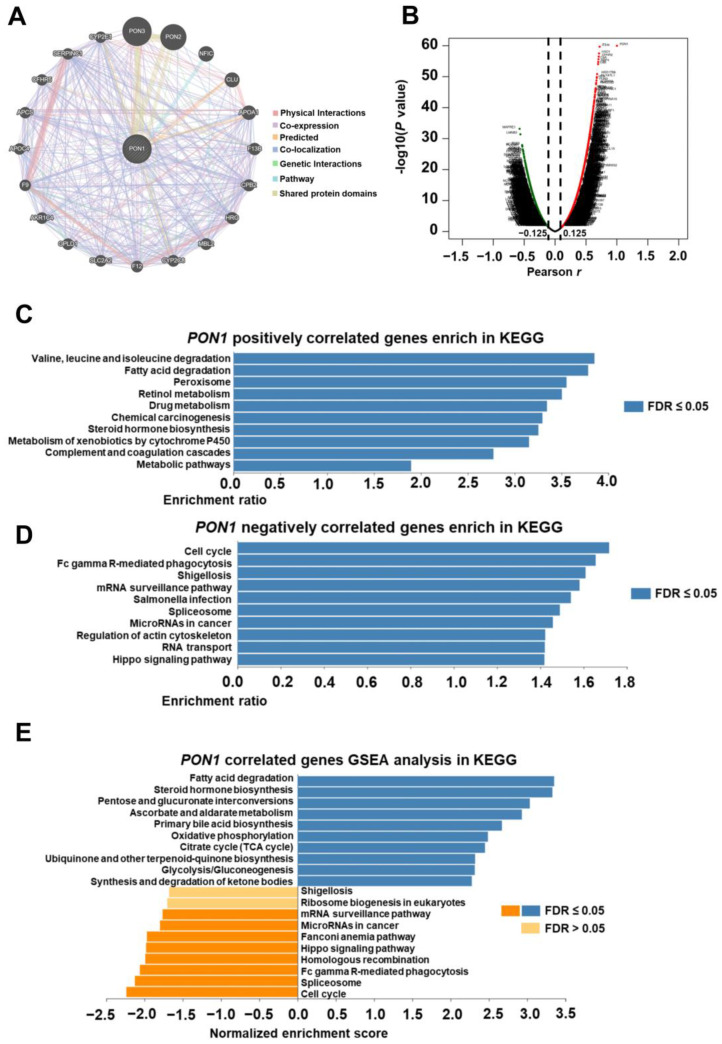
PPI network and correlation genes analysis of PON1. (**A**) Protein–protein interaction network for PON1 generated using GeneMANIA. (**B**) Volcano plot of genes correlated with *PON1* expression using LinkedOmics. Red dots, positively correlated genes (including *PON1*); green dots, negatively correlated genes. KEGG pathway enrichment analysis for genes positively (**C**) and negatively (**D**) correlated with *PON1*, and GSEA analysis for *PON1*-related gene sets (**E**).

A protein–protein interaction (PPI) network for PON1 was constructed using the GeneMANIA database, revealing that PON1 is embedded within a complex interaction network, with its most closely connected partners including its paralogs PON2 and PON3 (Figure 5A). To identify genes co-expressed with *PON1* on a genome-wide scale, correlation analysis was performed on TCGA_LIHC data. Applying thresholds of *p* < 0.05 and |Pearson *r*| > 0.125 yielded a total of 10,807 genes significantly correlated with *PON1* expression, comprising 3225 positively and 7582 negatively correlated genes (Figure 5B). Kyoto Encyclopedia of Genes and Genomes (KEGG) pathway analysis showed that genes positively correlated with *PON1* were significantly enriched in fatty acid degradation (*p* < 0.001) and peroxisome (*p* < 0.001) (Figure 5C). Conversely, genes negatively correlated with *PON1* showed strong enrichment in cell cycle (*p* < 0.001), spliceosome (*p* < 0.001), and RNA transport (*p* < 0.001) (Figure 5D). GSEA further validated and extended these observations (Figure 5E). Additionally, Gene Ontology Biological Process (GO-BP) enrichment analysis was conducted (Figure S2). Particularly, GO-BP results showed that genes positively correlated with *PON1* were significantly enriched in the lipid catabolic process (*p* < 0.001) (Figure S2A), and genes negatively correlated with *PON1* were predominantly enriched in the cell cycle process (*p* < 0.001) (Figure S2B).

### 3.5. Knockdown of PON1 Promotes the Malignant Phenotype of HCC Cells

**Figure 6:**
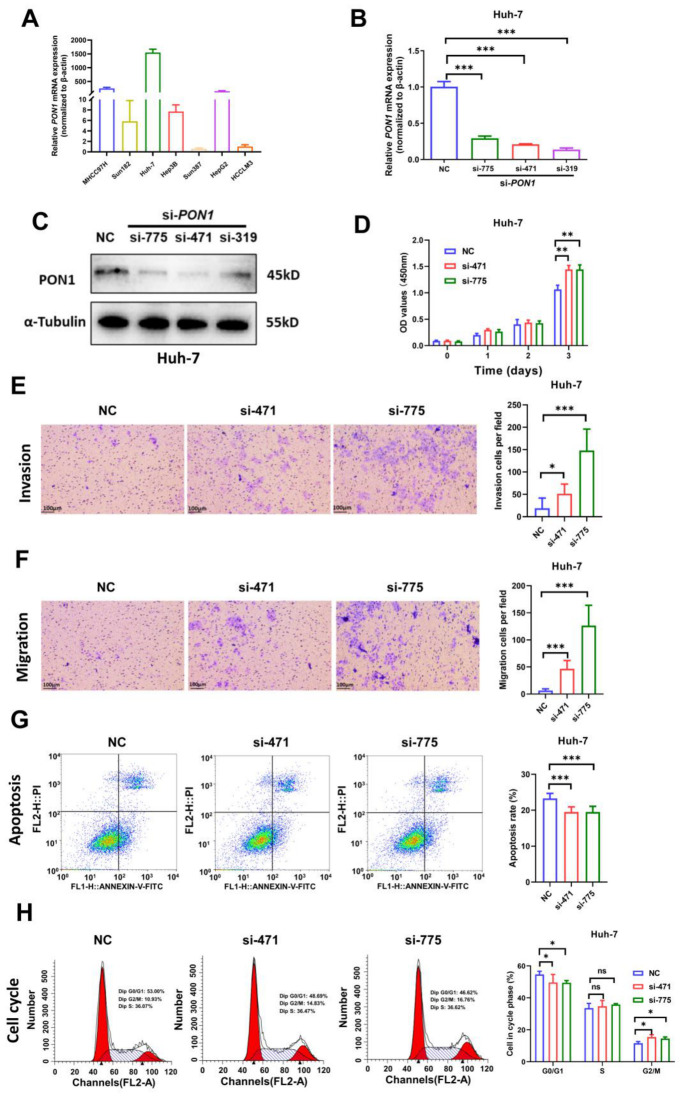
Knockdown of *PON1* enhanced malignant phenotypes of HCC cells. (**A**) qRT-PCR analysis of *PON1* mRNA expression in seven HCC cell lines. (**B**) qPCR detection of siRNA interference efficiency targeting *PON1*. (**C**) Western blotting detection of siRNA interference efficiency targeting *PON1*. Original western blots can be found at Supplementary Materials. The effect of siRNA knockdown of *PON1* on the proliferation (**D**), invasion (**E**) and migration (**F**), cell apoptosis (**G**) and cell cycle (**H**) of HCC cells in vitro. For (**B**,**D**–**H**), Student’s *t*-test. ns, not significant; * *p* < 0.05; ** *p* < 0.01; *** *p* < 0.001.

We used qRT-PCR to detect the mRNA expression of *PON1* in various liver cancer cell lines. The results showed that *PON1* was highly expressed in Huh-7 cells and lowly expressed in HCCLM3 and SNU387 cells (Figure 6A). To elucidate the role of *PON1* in HCC, *PON1* knockdown was performed in the Huh-7 cell line. We verified the interference efficiency of siRNA targeting the expression of *PON1*, and screened out two siRNAs (si-471 and si-775) that significantly reduced *PON1* mRNA and protein expression for further use in subsequent cell function experiments (Figure 6B,C). The CCK-8 assay demonstrated that *PON1* knockdown significantly enhanced the proliferative capacity of HCC cells (Figure 6D). The results of the transwell assay indicated that the migration and invasion abilities of HCC cells were significantly increased after *PON1* gene knockdown (Figure 6E,F). The apoptosis assay demonstrated that *PON1* knockdown markedly reduced the proportion of apoptotic cells (Figure 6G). Flow cytometry analysis of the cell cycle revealed that, compared with the control group, knockdown of *PON1* led to a significant increase in the proportion of cells in the G2/M phase and a decrease in the G0/G1 phase, suggesting that *PON1* knockdown promoted cell cycle progression of HCC cells (Figure 6H).

### 3.6. Overexpression of PON1 Inhibits the Malignant Phenotype of HCC Cells

**Figure 7:**
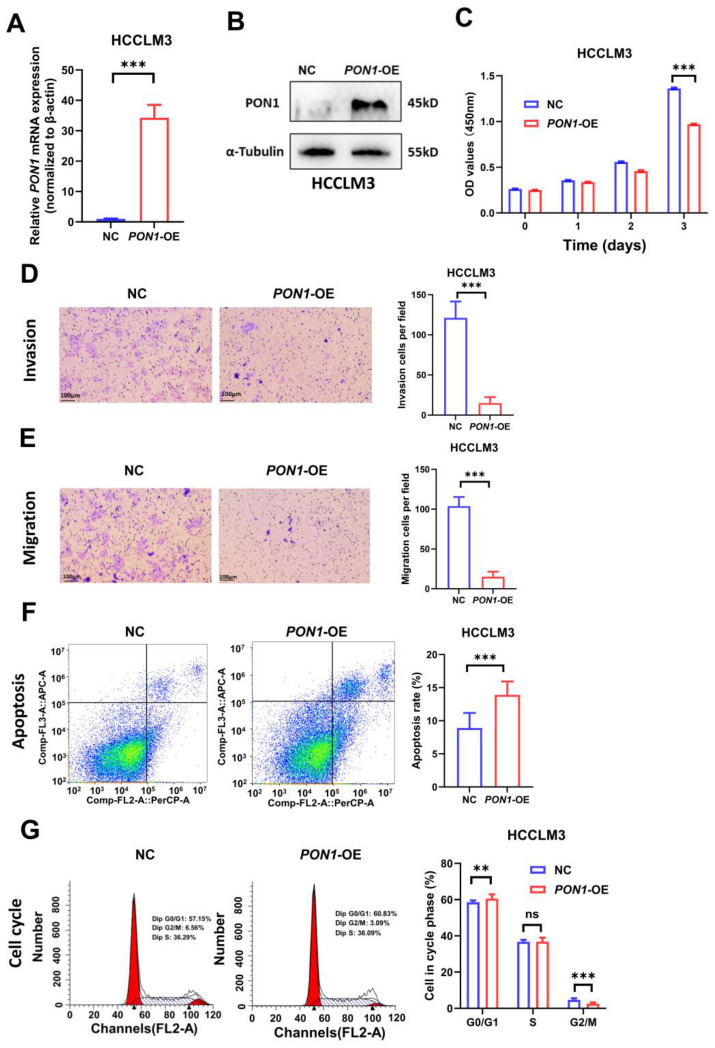
Overexpression of *PON1* inhibited malignant phenotypes of HCC cells. (**A**) qPCR detection of expression efficiency of stably overexpressed *PON1*. (**B**) Western blotting analysis of expression efficiency of stably overexpressed *PON1*. Original western blots can be found at Supplementary Materials. The effect of stably overexpressed *PON1* on the proliferation (**C**), invasion (**D**) and migration (**E**), cell apoptosis (**F**) and cell cycle (**G**) of HCC cells in vitro. For (**A**,**C**–**G**), Student’s *t*-test. ns, not significant; ** *p* < 0.01; *** *p* < 0.001.

We established stable overexpression of *PON1* in the HCCLM3 cell line and validated the overexpression efficiency by qRT-PCR (Figure 7A) and Western blot (Figure 7B). Subsequently, we employed the CCK-8 assay to investigate the proliferative capacity of HCC cells following *PON1* overexpression. The results showed that *PON1* overexpression significantly inhibited the proliferation of HCC cells (Figure 7C). Transwell assays revealed a striking diminishment in invasion and migration abilities upon *PON1* overexpression in HCCLM3 cells (Figure 7D,E). Flow cytometry analysis revealed that *PON1* overexpression increased the apoptosis rate of HCCLM3 cells (Figure 7F). In addition, *PON1* overexpression caused cell cycle arrest at the G0/G1 phase, resulting in a significant decrease in the proportion of cells entering the G2/M phase (Figure 7G). Overall, these results indicate that *PON1* plays a key role in suppressing the malignant progression of HCC cells.

### 3.7. PON1 Exerts Its Tumor-Suppressive Effect by Negatively Regulating the NOD-like Receptor Signaling Pathway

**Figure 8:**
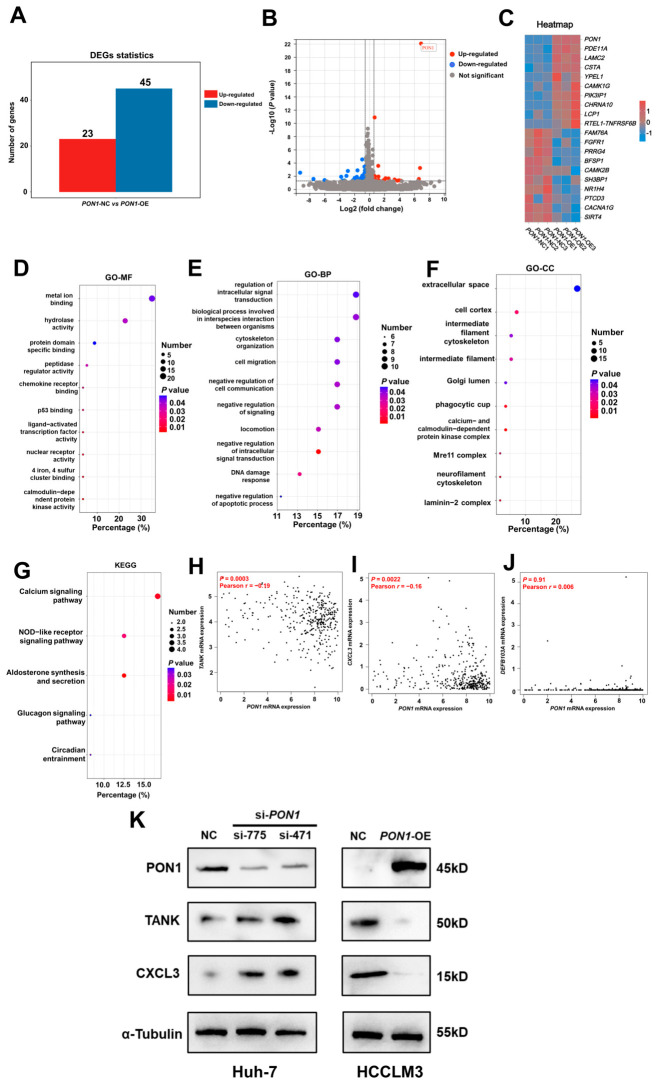
Overexpression of *PON1* suppressed the NOD-like receptor signaling pathway in HCC. (**A**) Bar chart for the statistics of DEGs. (**B**) Volcano plot of DEGs. (**C**) Heatmap of representative DEGs. GO enrichment analysis for Molecular Function (MF) (**D**), Biological Process (BP) (**E**), and Cellular Component (CC) (**F**) terms. (**G**) KEGG pathway analysis of DEGs. (**H**–**J**) Pearson correlation analysis between the mRNA expression of *PON1* and candidate target genes, *TANK*, *CXCL3*, and *DEFB103A* in the TCGA_LIHC. (**K**) Western blotting validation of PON1-mediated regulation of TANK and CXCL3 proteins. Original western blots can be found at Supplementary Materials.

To elucidate the molecular mechanisms underlying the tumor-suppressive function of *PON1*, we performed RNA-seq using *PON1*-stable overexpressing (PON1-OE) and negative control (NC) HCCLM3 cells, with thresholds set at |log_2_FC| > 0.585 and *p* < 0.05. The results identified a total of 68 differentially expressed genes (DEGs), comprising 23 upregulated and 45 downregulated genes (Figure 8A–C). GO enrichment analysis revealed that these DEGs were significantly associated with molecular functions (MF) including metal ion binding (*p* < 0.05) and hydrolase activity (*p* < 0.05) (Figure 8D). For biological processes (BP), the enriched terms were mainly related to negative regulation of intracellular signal transduction (*p* < 0.05) and cell migration (*p* < 0.05) (Figure 8E). For cellular components (CCs), significant enrichment was observed in terms such as the phagocytic cup (*p* < 0.05) (Figure 8F). KEGG pathway analysis further demonstrated significant enrichment of DEGs in pathways such as calcium signaling (*p* < 0.01) and the NOD-like receptor signaling pathway (*p* < 0.05) (Figure 8G). Given the central role of the NOD-like receptor pathway in sensing intracellular danger signals, regulating inflammatory and immune responses, and modulating apoptosis [30], this pathway emerged as a plausible mediator of the tumor-suppressive effects of *PON1*. RNA-seq results revealed significant downregulation of the NOD-like receptor pathway key molecules *TANK*, *CXCL3*, and *DEFB103A* in the *PON1* overexpression group. Correlation analysis of TCGA_LIHC data revealed a negative correlation between *PON1* expression and the mRNA levels of *TANK* (Pearson *r* = −0.19, *p* = 0.0003) and *CXCL3* (Pearson *r* = −0.16, *p* = 0.0022). However, no significant correlation was observed between *PON1* and *DEFB103A* (Pearson *r* = 0.006, *p* = 0.91) (Figure 8H–J). Western blotting was performed to verify the above results, and it was found that knockdown of PON1 expression upregulated the protein expression of TANK and CXCL3. Moreover, overexpression of PON1 also downregulated the expression of TANK and CXCL3 (Figure 8K). These results indicate that PON1 suppresses the expression of TANK and CXCL3, key molecules in the NOD-like receptor signaling pathway, thereby exerting tumor-suppressive functions in HCC.

## 4. Discussion

This study presents a comprehensive elucidation of PON1 as a potential tumor suppressor in HCC. Through multiple independent databases, *PON1* was identified as a significantly downregulated gene and was shown to have independent prognostic significance. The pronounced enrichment of PON1 in normal hepatocytes suggested that PON1 may play a core role in maintaining liver-specific metabolic homeostasis, including lipid and xenobiotic metabolism, as well as antioxidant defense [4,5]. In HCC, downregulation of PON1 expression may indicate the loss of tumor-suppressive function in hepatocytes, thereby leading to a lack of protective signals in the microenvironment.

PON1 is a liver-derived antioxidant enzyme associated with HDL and exhibits lactonase, arylesterase and paraoxonase activities [4,5]. Through these functions, it detoxifies exogenous compounds, protects low-density lipoprotein from oxidation [31] and removes pro-inflammatory lactones, thereby maintaining HDL integrity and suppressing atherosclerosis [5]. Consequently, PON1 serves as both a key player in oxidative stress-related diseases and a potential early biomarker. Classically, PON1 is viewed as a secreted enzyme whose proposed systemic anti-cancer effects occur upon hepatic synthesis and HDL-associated circulation. In this context, it degrades pro-inflammatory mediators like oxidized phospholipids, thereby reducing the oxidative stress and inflammatory tone that foster neoplastic initiation and progression [4,5].

Previous bioinformatics analyses have shown that *PON1* served as a prognostic biomarker and a preoperative predictor of microvascular invasion in HCC [10,11]. Subsequent bioinformatics analyses further linked low *PON1* expression to unfavorable prognosis [12]. Collectively, these studies have firmly established the clinical relevance of *PON1*. Despite these advances, several significant limitations remain unaddressed. A systematic bioinformatics analysis of *PON1* that integrates expression data and clinical features across large cohorts is still lacking, leaving its expression landscape and prognostic value incompletely characterized. In the current study, we analyzed the expression and clinical characteristics of *PON1* through systematic bioinformatics data analysis. Based on multiple independent databases, including TCGA, ICGC, and GEO, we identified *PON1* as an important favorable prognostic factor in HCC. Furthermore, our findings demonstrate that decreased *PON1* expression contributes to the malignant progression of HCC cells, thereby establishing PON1 as a promising prognostic biomarker.

Moreover, Lu et al. demonstrated that PON1 suppressed HCC by promoting VHL-mediated HIF-1α degradation, reducing lactate production, and limiting regulatory T cell infiltration [13]. Nevertheless, the precise functional mechanisms by which PON1 directly suppresses malignant phenotypes of HCC cells remain poorly defined. Therefore, we performed PON1 functional experiments based on HCC cells to investigate the effects of PON1 on cellular biological functions, including proliferation, migration, invasion, apoptosis, and cell cycle progression. In vitro experiments validated that PON1 directly attenuated hallmark malignant phenotypes of HCC cells, encompassing proliferation, migration, invasion, and evasion of apoptosis, as well as inhibiting the cell cycle progression of liver cancer cells.

Lu et al. provided initial mechanistic insights [13]. However, whether PON1 participates in other regulatory pathways (such as inflammation-related signaling pathways) remains completely unknown. In order to conduct a further study on this issue, we employed protein–protein interaction network analysis and the results of the pathway enrichment showed that high *PON1* expression is associated with activation of catabolic pathways, including fatty acid degradation and branched-chain amino acid breakdown. In addition, low *PON1* expression correlates with enrichment of cell cycle and DNA replication. These findings suggest that *PON1* may function as a hub molecule linking the metabolism and proliferation of HCC cells. This interpretation aligns with the known biological functions of PON1, whose esterase/lactonase activity has been implicated in the regulation of lipid metabolites and oxidative stress [5,32]. Future studies directly measuring metabolic alterations of HCC cells following *PON1* deletion will be essential to experimentally validate this hypothesis. As a secreted protein, PON1 could potentially attenuate tumor-associated chronic inflammation through hydrolysis of oxidized phospholipids [4,5,32]. Additionally, it may modulate immune cell recruitment by influencing the chemokine network [33,34].

More importantly, our RNA-seq and experimental data identified a previously unrecognized role of *PON1* in negatively regulating the NOD-like receptor signaling pathway through downregulation of *TANK* and *CXCL3*. The NOD-like receptor (NLR) signaling pathway functions as an innate immune sensor that recognizes pathogen-associated and damage-associated molecular patterns [35]. Upon activation, most NLR signaling molecules trigger NF-κB through the adaptor protein RIPK2, leading to the production of pro-inflammatory cytokines and chemokines [36,37]. TANK serves as a signal mediator of NF-κB activation. It acts as a co-inducer with TRAF2 to synergistically activate NF-κB, thereby promoting inflammatory responses [38]. CXCL3 is a downstream chemokine whose promoter contains an NF-κB binding site [39], and its transcription is directly induced by NF-κB upon NLR activation [40]. This finding reveals a novel mechanism by which PON1 exerts its tumor-suppressive effects via inflammatory pathways.

Several limitations of this study should be acknowledged. First, although complementary loss- and gain-of-function experiments validated the regulatory effect of PON1 on malignant phenotypes and its impact on TANK and CXCL3 expression, the precise molecular mechanism through which PON1 modulates these downstream targets remains to be elucidated. Second, the current findings are primarily based on cell line models, whereas in vivo validation of PON1 in the malignant progression and molecular mechanism of HCC cells requires further confirmation. Future studies utilizing conditional knockout or transgenic overexpression mouse models will be essential to establish the functional role of PON1 in physiologically relevant settings.

## 5. Conclusions

In summary, our study preliminarily confirms that PON1 may serve as a potential tumor suppressor in HCC. Downregulation of *PON1* promotes proliferation, invasion, migration, cell cycle progression, and inhibits apoptosis of HCC cells. Furthermore, *PON1* can serve as an independent prognostic marker for HCC. Mechanistically, PON1 negatively regulates the NOD-like receptor pathway by inhibiting TANK and CXCL3. These findings position PON1 as both a robust prognostic biomarker and a promising therapeutic target for HCC.

## Figures and Tables

**Table 1:** Univariate and multivariate Cox regression analysis for overall survival in TCGA_LIHC.

Variable		Univariate		Multivariate	
		HR	*p* Value	HR	*p* Value
	*PON1*	0.858 (0.798–0.921)	0.000	0.846 (0.777–0.921)	0.000
Clinical characteristics					
	Age	1.010 (0.996–1.025)	0.174	1.013 (0.999–1.028)	0.071
	Gender	0.776 (0.531–1.132)	0.188	1.118 (0.743–1.682)	0.593
	Grade	1.133 (0.881–1.457)	0.330	1.127 (0.854–1.488)	0.398
	Stage	1.680 (1.369–2.062)	0.000	1.600 (1.291–1.983)	0.000
Immune cell infiltration					
	B cell	0.878 (0.580–1.329)	0.538	0.896 (0.461–1.738)	0.744
	CD4^+^ T cell	1.300 (0.799–2.115)	0.291	1.136 (0.535–2.411)	0.739
	CD8^+^ T cell	0.798 (0.475–1.339)	0.393	0.441 (0.205–0.949)	0.036
	Macrophage	1.058 (0.932–1.200)	0.386	0.984 (0.828–1.168)	0.851
	Neutrophil	2.130 (0.930–4.879)	0.074	2.692 (0.798–9.080)	0.110

HR, hazard ratio; *PON1*, paraoxonase 1. Data in parentheses are 95% confidence intervals.

## Data Availability

The original contributions presented in the study are included in the article/Supplementary Materials. Data for the RNA-seq are accessible from the Gene Expression Omnibus under Accession No. GSE322594.

## Supplementary Materials

The following supporting information can be downloaded at https://www.mdpi.com/article/10.3390/biom16060774/s1, Figure S1: RNA and protein expression of PON1 in CCLE; Figure S2: GO-BP enrichment analysis of PON1 correlated genes; Table S1: VIF value of PON1 expression, clinical characteristics and immune cells TCGA_LIHC; File S1: Original Western blot images.

## Abbreviations

The following abbreviations are used in this manuscript:

ANOVA	Analysis of variance
CCLE	Cancer Cell Line Encyclopedia
CHOL	Cholangiocarcinoma
CNV	Copy number variations
CPTAC	Clinical Proteomic Tumor Analysis Consortium
DCs	Dendritic cells
GEO	Gene Expression Omnibus
GO	Gene Ontology
GSEA	Gene set enrichment analysis
HCC	Hepatocellular carcinoma
HDL	High-density lipoprotein
HNSC	Head and neck squamous cell carcinoma
HPA	Human Protein Atlas
HR	Hazard ratio
ICGC	International Cancer Genome Consortium
KEGG	Kyoto Encyclopedia of Genes and Genomes
LIHC	Liver hepatocellular carcinoma
LUAD	Lung adenocarcinoma
OS	Overall survival
PON1	Paraoxonase 1
scRNA-seq	Single-cell RNA sequencing
TCGA	The Cancer Genome Atlas
TPM	Transcripts per million
VIF	Variance inflation factor
