# Supplementary material for: Paraoxonase 1 Suppresses Hepatocellular Carcinoma Progression by Modulating the NOD-like Receptor Signaling Pathway
Source: Biomolecules. 2026 May 25;16(6):774. doi: 10.3390/biom16060774 (PMC13297327; doi:10.3390/biom16060774)
Supplement: Supplementary file 1 [file biomolecules-16-00774-s001.zip › Table S1.pdf]

Table S1. VIF value of *PON1* expression, clinical characteristics and immune cells TCGA_LIHC.

**Variable**		**VIF**
	PON1	1.240
Clinical characteristics		
	Age	1.046
	Gender	1.083
	Grade	1.079
	Stage	1.097
Immune cell infiltration		
	B cell	3.767
	$CD4^+$ T cell	2.174
	$CD8^+$ T cell	4.694
	DCs	7.092
	Macrophage	1.881
	Neutrophil	2.251

DCs, dendritic cells; *PON1*, paraoxonase 1; VIF, variance inflation factor
