# Supplementary material for: Paraoxonase 1 Suppresses Hepatocellular Carcinoma Progression by Modulating the NOD-like Receptor Signaling Pathway
Source: Biomolecules. 2026 May 25;16(6):774. doi: 10.3390/biom16060774 (PMC13297327; doi:10.3390/biom16060774)
Supplement: Supplementary file 1 [file biomolecules-16-00774-s001.zip › Figure S1.pdf]

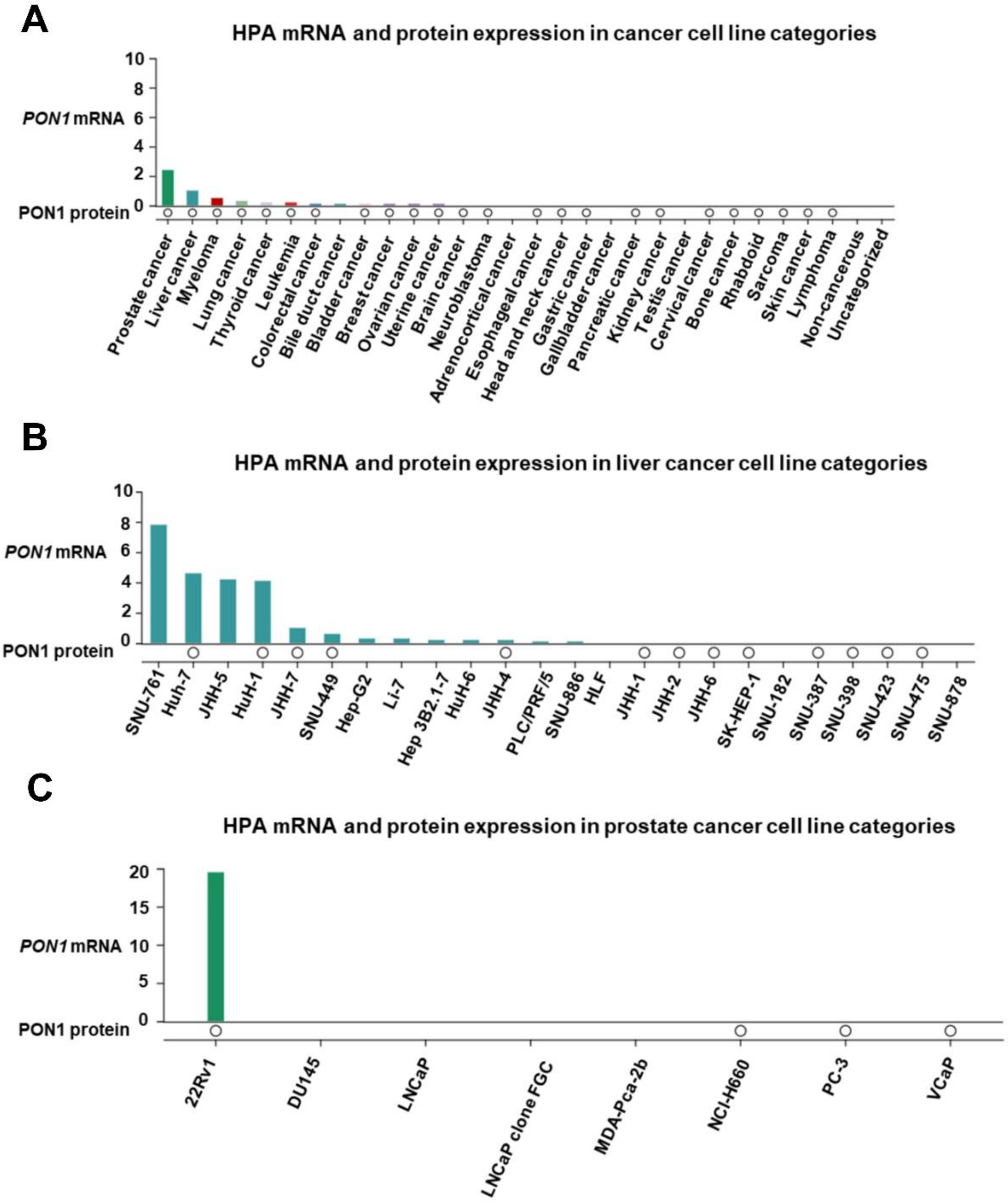

Figure S1. RNA and protein expression of PON1 in CCLE. (A) the mRNA and protein expression of PON1 across cancer cell line categories. RNA expression of *PON1* and protein expression of PON1 in liver cancer (B) and prostate cancer (C) cell lines.
