# Supplementary material for: Paraoxonase 1 Suppresses Hepatocellular Carcinoma Progression by Modulating the NOD-like Receptor Signaling Pathway
Source: Biomolecules. 2026 May 25;16(6):774. doi: 10.3390/biom16060774 (PMC13297327; doi:10.3390/biom16060774)
Supplement: Supplementary file 1 [file biomolecules-16-00774-s001.zip › Figure S2.pdf]

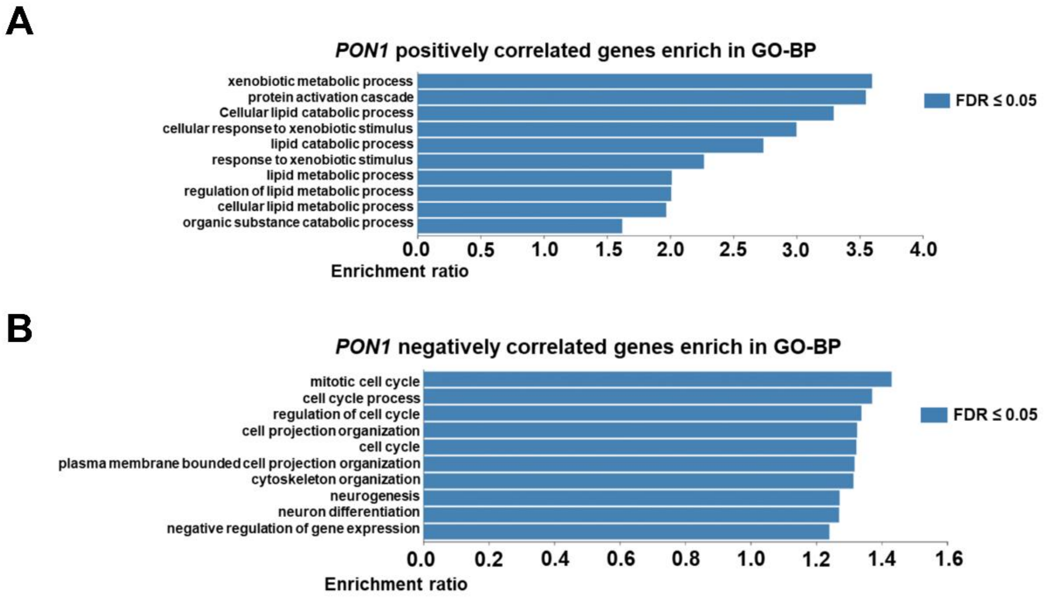

Figure S2. GO-BP enrichment analysis of *PON1* correlated genes. GO-BP enrichment analysis for genes positively (A) and negatively (B) correlated with *PON1* in Biological Process.
