# Supplementary figures and images for: Paraoxonase 1 Suppresses Hepatocellular Carcinoma Progression by Modulating the NOD-like Receptor Signaling Pathway
Source: Biomolecules. 2026 May 25;16(6):774. doi: 10.3390/biom16060774 (PMC13297327; doi:10.3390/biom16060774)

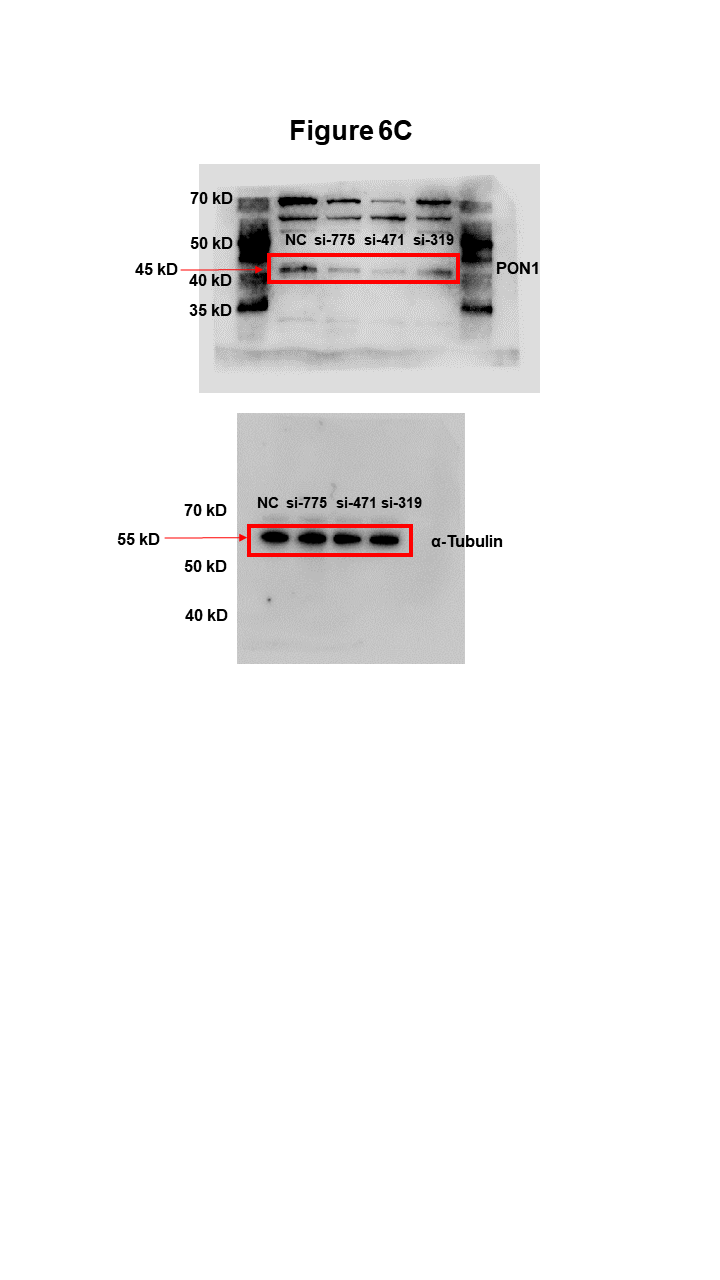

Supplement: Supplementary file 1 [file biomolecules-16-00774-s001.zip › File S1/File S1-1.TIF]

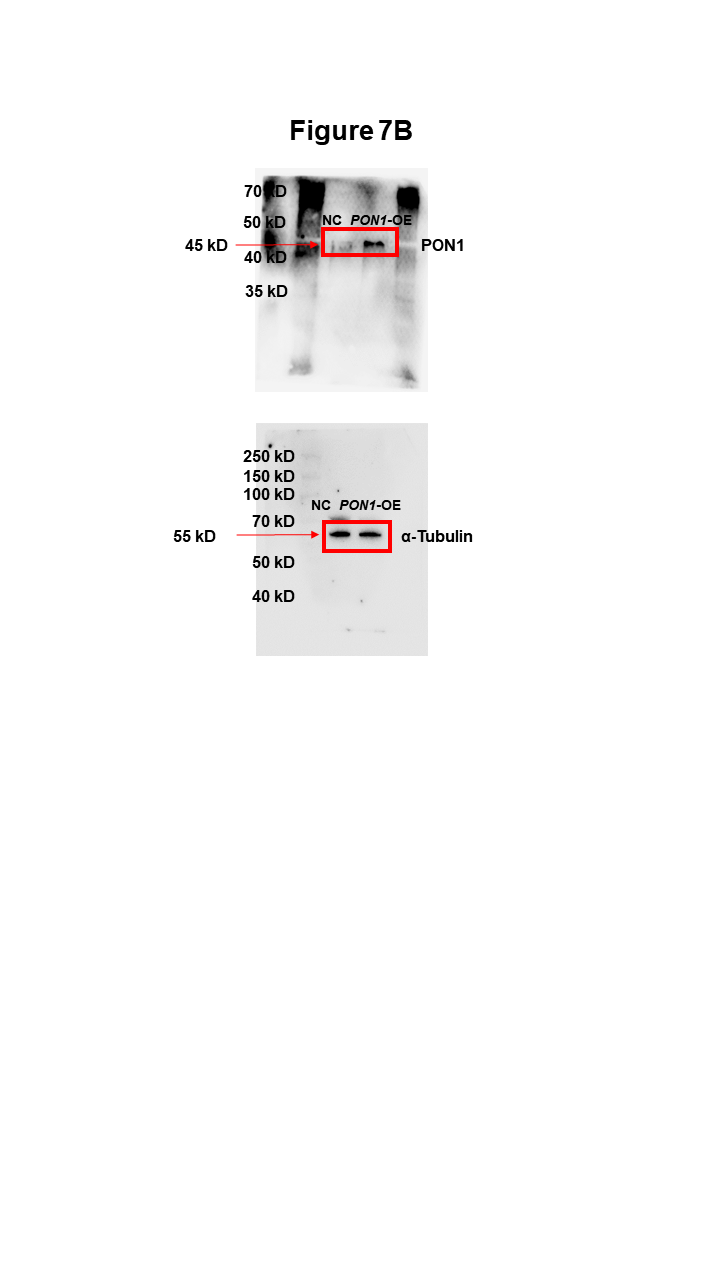

Supplement: Supplementary file 1 [file biomolecules-16-00774-s001.zip › File S1/File S1-2.TIF]

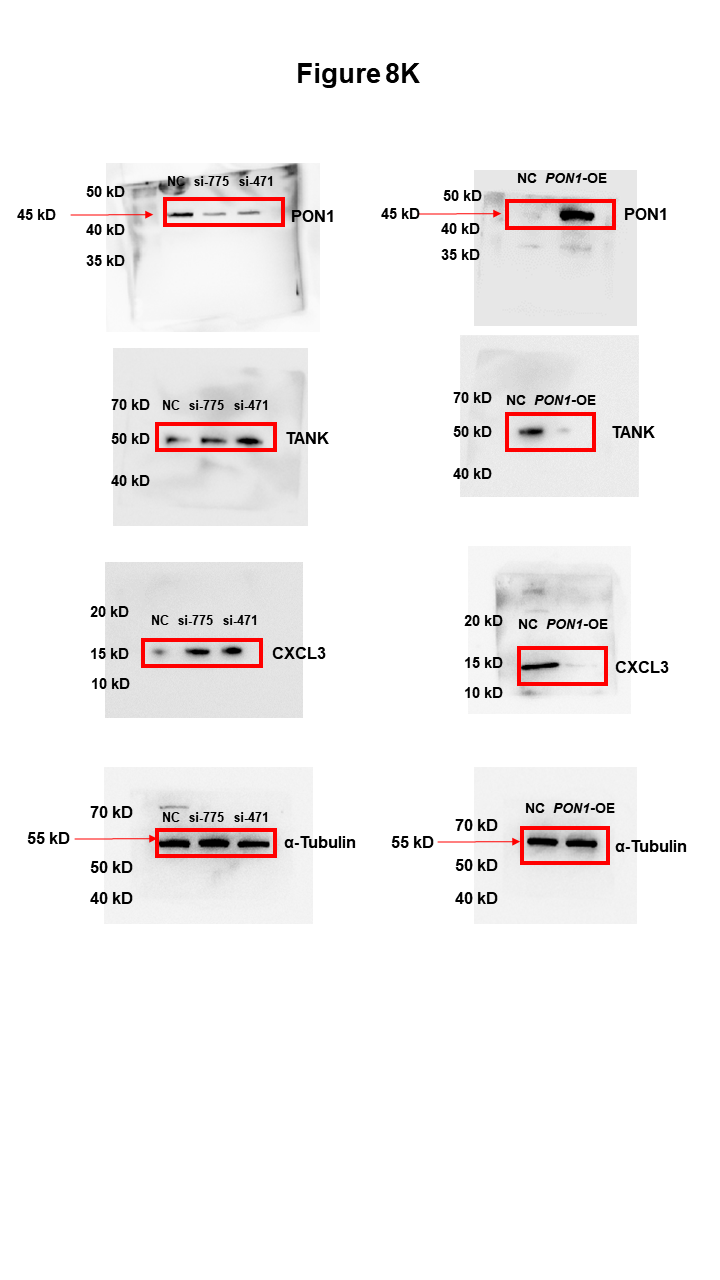

Supplement: Supplementary file 1 [file biomolecules-16-00774-s001.zip › File S1/File S1-3.TIF]
